# Tinostamustine (EDO-S101), an Alkylating Deacetylase Inhibitor, Enhances the Efficacy of Daratumumab in Multiple Myeloma by Upregulation of CD38 and NKG2D Ligands

**DOI:** 10.3390/ijms25094718

**Published:** 2024-04-26

**Authors:** Andrea Díaz-Tejedor, Javier Rodríguez-Ubreva, Laura Ciudad, Mauro Lorenzo-Mohamed, Marta González-Rodríguez, Bárbara Castellanos, Janet Sotolongo-Ravelo, Laura San-Segundo, Luis A. Corchete, Lorena González-Méndez, Montserrat Martín-Sánchez, María-Victoria Mateos, Enrique M. Ocio, Mercedes Garayoa, Teresa Paíno

**Affiliations:** 1 Centro de Investigación del Cáncer-Instituto de Biología Molecular y Celular del Cáncer (CIC-IBMCC), Universidad de Salamanca, Consejo Superior de Investigaciones Científicas (CSIC), 37007 Salamanca, Spain; adiaz062@usal.es (A.D.-T.); lorenzomohamed.mauro@usal.es (M.L.-M.); marta.gonzalez04@usal.es (M.G.-R.); barbaracas@usal.es (B.C.); sotolongojanet@usal.es (J.S.-R.); duckey@usal.es (L.S.-S.); lcorchetesanchez@mgh.harvard.edu (L.A.C.); lgonzalez@usal.es (L.G.-M.); monseratt@usal.es (M.M.-S.); mvmateos@usal.es (M.-V.M.); mgarayoa@usal.es (M.G.); 2 Servicio de Hematología, Complejo Asistencial Universitario de Salamanca, Instituto de Investigación Biomédica de Salamanca (IBSAL), 37007 Salamanca, Spain; 3 Epigenetics and Immune Disease Group, Josep Carreras Research Institute (IJC), 08916 Badalona, Spain; jrodriguez@carrerasresearch.org (J.R.-U.); lciudad@carrerasresearch.org (L.C.); 4 Centro de Investigación Biomédica En Red de Cáncer (CIBERONC, CB16/12/00233), Instituto de Salud Carlos III (ISCIII), 28029 Madrid, Spain; 5 Departamento de Medicina, Universidad de Salamanca, 37007 Salamanca, Spain; 6 Hospital Universitario Marqués de Valdecilla (IDIVAL), Universidad de Cantabria, 39008 Santander, Spain; ocioem@unican.es; 7 Departamento de Fisiología y Farmacología, Universidad de Salamanca, 37007 Salamanca, Spain

**Keywords:** multiple myeloma, daratumumab, tinostamustine, immunotherapy

## Abstract

Multiple myeloma is a malignancy characterized by the accumulation of malignant plasma cells in bone marrow and the production of monoclonal immunoglobulin. A hallmark of cancer is the evasion of immune surveillance. Histone deacetylase inhibitors have been shown to promote the expression of silenced molecules and hold potential to increase the anti-MM efficacy of immunotherapy. The aim of the present work was to assess the potential effect of tinostamustine (EDO-S101), a first-in-class alkylating deacetylase inhibitor, in combination with daratumumab, an anti-CD38 monoclonal antibody (mAb), through different preclinical studies. Tinostamustine increases CD38 expression in myeloma cell lines, an effect that occurs in parallel with an increment in CD38 histone H3 acetylation levels. Also, the expression of MICA and MICB, ligands for the NK cell activating receptor NKG2D, augments after tinostamustine treatment in myeloma cell lines and primary myeloma cells. Pretreatment of myeloma cell lines with tinostamustine increased the sensitivity of these cells to daratumumab through its different cytotoxic mechanisms, and the combination of these two drugs showed a higher anti-myeloma effect than individual treatments in ex vivo cultures of myeloma patients’ samples. In vivo data confirmed that tinostamustine pretreatment followed by daratumumab administration significantly delayed tumor growth and improved the survival of mice compared to individual treatments. In summary, our results suggest that tinostamustine could be a potential candidate to improve the efficacy of anti-CD38 mAbs.

## 1. Introduction

Multiple myeloma (MM), the second most common hematological malignancy, is characterized by the accumulation of malignant plasma cells in bone marrow (BM) and the production of monoclonal immunoglobulin [1]. CD38 is a type II transmembrane glycoprotein highly expressed on malignant plasma cells at all stages of the disease. Considering this overexpression, CD38 emerged as an attractive immunotherapeutic target for MM a decade ago [2], daratumumab being the first anti-CD38 monoclonal antibody (mAb) approved for MM patients in 2015 [3]. Daratumumab is a human IgG1 mAb currently used in monotherapy or in combination with standard-of-care treatments to treat both relapsed and refractory MM (RRMM) [4,5,6,7] or newly diagnosed patients [8,9,10]. Although anti-CD38 therapy has generally shown to be highly effective for MM treatment, there are still differences in its efficacy among patients [11], and despite initial good responses, some patients eventually relapse [12].

Daratumumab exerts its anti-myeloma effect through immune-mediated mechanisms (e.g., antibody-dependent cellular cytotoxicity (ADCC), complement-mediated cytotoxicity (CDC) and antibody-dependent cellular phagocytosis (ADCP)) [13,14]. Additionally, this mAb induces apoptosis via Fcγ receptor-mediated crosslinking [15]. Also, daratumumab promotes immunomodulatory effects by decreasing the absolute cell count of immunosuppressive regulatory T cells, myeloid-derived suppressor cells and B regulatory cells, consequently increasing the number and activation of cytotoxic T cells [16]. Among other factors, response to daratumumab is associated with CD38 levels on tumor cells [11,17]. Therefore, agents able to increase CD38 may be good candidates to be combined with daratumumab.

Recently, epigenetic drugs such as DNA methyltransferase inhibitors (DNMTis) and histone deacetylase inhibitors (HDACis) were shown to upregulate CD38 expression, thus augmenting the anti-myeloma effect of daratumumab [18,19,20]. Furthermore, it was observed, not only in MM but also in other tumors, that epigenetic drugs increase the expression of immunologically relevant molecules, for example, ligands for activating NK cell receptors, MHC class I and II proteins, and costimulatory molecules. This increases the immunogenicity of tumor cells and renders them more susceptible to the attack by immune cells [21,22,23,24], thereby enhancing the effects of immunotherapies [25]. Tumor cells, including myeloma cells, are known to express ligands for activating NK cell receptors such as the MHC I-related molecules MICA/B and ULBPs, which are both NKG2D ligands [26]. Another activating receptor involved in NK cell-mediated tumor cell killing is DNAM-1, which recognizes Nectin-2 (Nec-2, CD112) and the poliovirus receptor (PVR, CD155) ligands [27]. Specifically, it has been demonstrated that HDACis are able to upregulate the expression of MICA/B, ULPBs, Nectin-2 and/or PVR in MM and other tumors [24,28,29]. In addition to epigenetic agents, other drugs, for example, alkylating agents and proteasome inhibitors also upregulate DNAM-1 and NKG2D ligands in MM cells [26].

Tinostamustine is a first-in-class alkylating deacetylase inhibitor with potent inhibitory activity against both class I and II HDACs [30]. Previously, our group reported the potent anti-myeloma activity of tinostamustine, as a single agent and in combination with bortezomib, in preclinical models [31]. The anti-tumor efficacy of tinostamustine has also been demonstrated in T-cell leukemia [32], acute myeloid leukemia [33] and brain tumors [34,35]. Moreover, tinostamustine is currently being evaluated in monotherapy in phase I/II clinical trials for the treatment of advanced solid tumors and relapsed/refractory hematologic malignancies, including RRMM (NCT03345485, NCT02576496); its efficacy is also being assessed in combination with other agents in glioblastoma and advanced melanoma (NCT03452930, NCT03903458). However, the role of this drug in combination with immunotherapies for multiple myeloma has not yet been explored.

Here, we assessed the effect of tinostamustine on the expression of CD38 and ligands for activating NK cell receptors in MM cells and subsequently explored its potential to enhance the anti-myeloma effect of daratumumab in preclinical models.

## 2. Results

### 2.1. Tinostamustine Increases CD38 Expression in Myeloma Cells

**Figure 1:**
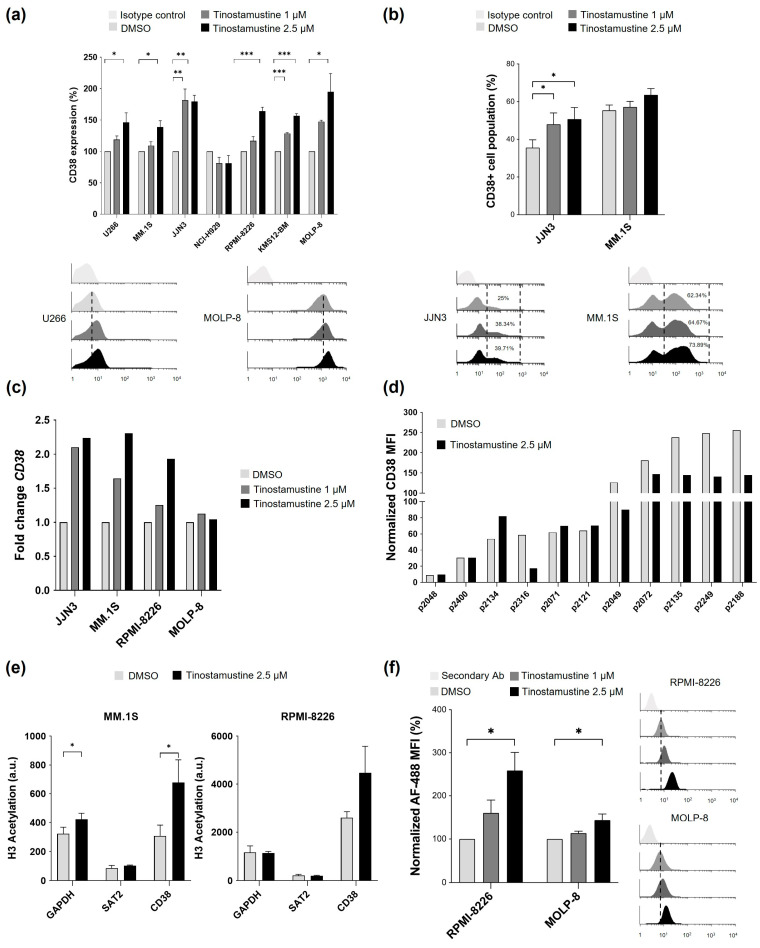
Evaluation of the effect of tinostamustine on CD38 expression. (**a**) Upper panel: normalized CD38 mean fluorescence intensity (MFI) determined by flow cytometry (FCM) after treatment of myeloma cell lines with tinostamustine (48 h). DMSO-treated cells are 100%. Lower pannel: representative histograms. (**b**) Upper panel: percentage of CD38^+^ cell population determined by FCM in JJN3 and MM.1S after tinostamustine treatment (48 h). Lower pannel: representative histograms. (**c**) *CD38* mRNA levels determined by RT-qPCR after incubation of myeloma cell lines with tinostamustine (MOLP-8, 6 h; other cell lines, 36 h). Results show the fold change between tinostamustine-treated and DMSO-treated cells after normalization with GAPDH (average of three experiments). (**d**) Normalized CD38 MFI determined by FCM on patients’ myeloma cells after ex vivo treatment with tinostamustine (48 h). (**e**) ChIP signal of histone H3 acetylation (AcH3) in the *CD38* gene. Chromatin isolated from MM.1S and RPMI-8226 cell lines after tinostamustine treatment (48 h) was immunoprecipitated using an anti-acetyl histone H3 antibody (AcH3) or an anti-IgG as a negative control. The GAPDH gene was used as a positive control for AcH3, and the SAT2 gene was used as a negative control. The signal from immunoprecipitated DNA relative to the signal of the ChIP input is shown. (**f**) Left: normalized MFI determined by FCM of anti-human-IgG1-AlexaFluor488 bound to daratumumab-coated myeloma cell lines after pretreatment with tinostamustine (48 h). Right: representative histograms. In (**a**,**b**,**e**,**f**), each bar shows the mean ± SEM (*n* = 3). Statistical differences were evaluated by one-way ANOVA followed by Tukey’s HSD post hoc test (* *p* < 0.05, ** *p* < 0.01 and *** *p* < 0.001). In (**a**,**b**,**f**) dashed lines are included to show the increase in fluorescence in tinostamustine-treated vs. DMSO-treated cells.

The effect of tinostamustine on CD38 expression was evaluated by flow cytometry (FCM) in seven MM cell lines with different basal expression levels of this protein (Figure S1). Treatment with tinostamustine (1 and 2.5 μM, 48 h) increased CD38 surface expression in the viable population in all cell lines except for NCI-H929 (Figure 1a). It should be highlighted that this effect was observed both in cell lines with low basal levels of CD38 (e.g., U266) and in those with high basal expression (e.g., RPMI-8226 and MOLP-8). Interestingly, in those MM cell lines with heterogeneous CD38 surface expression, that is, with a CD38^+^ and a CD38^−/low^ population (JJN3 and MM.1S), treatment with tinostamustine significantly increased the percentage of cells in the first population in the JJN3 cell line, and a similar tendency was observed for MM.1S cell line (Figure 1b). Note that the percentage of viable cells after treatment with the highest dose of tinostamustine ranged between 25.6% for the most sensitive cell line (MOLP-8) and 76.8% for the least sensitive one (JJN3) (Figure S2). For subsequent experiments, JJN3, MM.1S, RPMI-8226 and MOLP-8 cell lines were chosen due to their different CD38 basal expression levels and sensitivity to tinostamustine.

The increase in CD38 surface expression observed by FCM was confirmed by RT-qPCR in MM.1S, JJN3 and RPMI-8226 cell lines; however, in MOLP-8 cells, which show high cytotoxicity to tinostamustine (Figure S2), these results were not observed (Figure 1c). CD38 expression was further examined by FCM in malignant plasma cells from cultures of 11 MM patients’ bone marrow samples treated ex vivo with tinostamustine (2.5 μM) for 48 h. As shown in Figure 1d, tinostamustine (2.5 μM) slightly increased CD38 expression in primary myeloma cells in 3 out of 11 patients analyzed (p2134, p2071 and p2121), corresponding to some of those with low basal antigen expression, but this effect was not observed in any of the patients with high CD38 basal expression.

Since tinostamustine has potent HDAC inhibitory activity [30], next, we investigated if levels of histone acetylation were associated with the increased CD38 expression observed in MM cell lines. For this purpose, we performed a ChIP-qPCR analysis of the *CD38* gene in MM.1S and RPMI-8226 cell lines after treatment with DMSO or tinostamustine (2.5 μM), comparing the acetylation status of histone H3. In MM.1S cells, histone H3 acetylation levels of *CD38* were significantly increased by tinostamustine in comparison to DMSO, and a similar trend was observed with RPMI-8226 cells, although statistical significance was not reached (Figure 1e).

Finally, we assessed whether the increase in CD38 antigen expression promoted by tinostamustine was able to improve the binding of daratumumab to myeloma cells. As Figure 1f shows, pretreatment with tinostamustine (2.5 μM) significantly enhanced the binding of daratumumab in RPMI-8226 and MOLP-8 cell lines compared with DMSO controls.

### 2.2. Tinostamustine Increases the Expression of MICA and MICB, Ligands for the Activating NK Cell Receptor NKG2D, in Myeloma Cell Lines and Malignant Plasma Cells from Myeloma Patients

**Figure 2:**
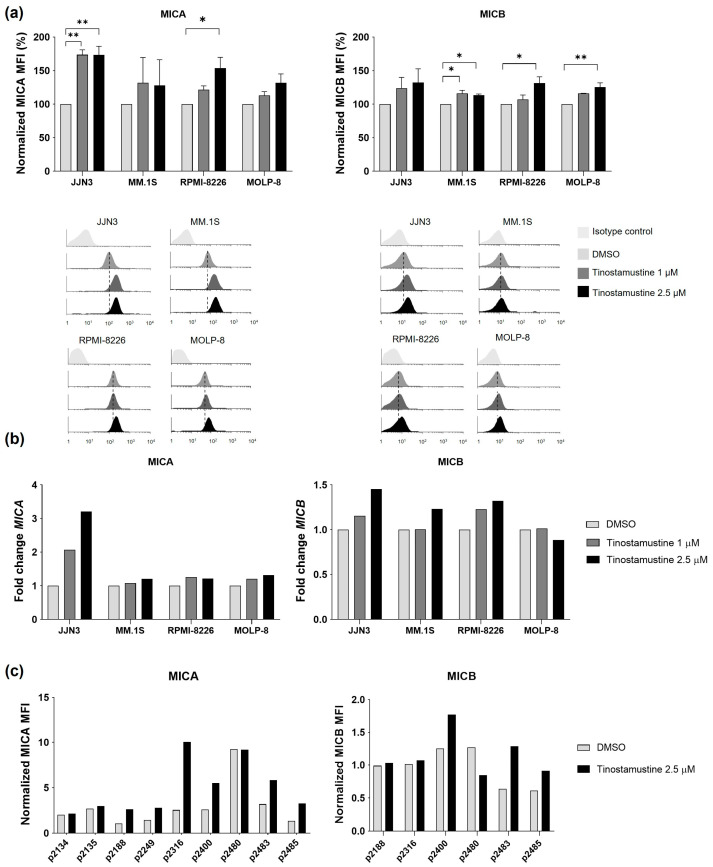
Effect of tinostamustine on the expression of MICA and MICB. (**a**) Upper panels: normalized MFI determined by FCM of MICA and MICB on myeloma cell lines after treatment with tinostamustine (48 h). Each bar shows the mean ± SEM (*n* = 3). Statistically significant differences were evaluated by one-way ANOVA followed by Tukey’s HSD post hoc test (* *p* < 0.05 and ** *p* < 0.01). Lower panels: representative histograms. (**b**) *MICA* and *MICB* mRNA levels determined by RT-qPCR after treatment with tinostamustine (MOLP-8, 6 h; other cell lines, 36 h). Results show the fold change between tinostamustine-treated and DMSO-treated cells after normalization with GAPDH (average of three experiments). (**c**) Normalized MFI of MICA and MICB determined by FCM on myeloma cells from patients after ex vivo treatment with tinostamustine (48 h). In (**a**) dashed lines are included to show the increase in fluorescence in tinostamustine-treated vs. DMSO-treated cells.

Since HDACis and alkylating agents have been reported to modulate the expression of ligands for activating NK cell receptors [24,26,28,29], we investigated the effect of tinostamustine on the expression of NKG2D and DNAM-1 ligands in several MM cell lines. Tinostamustine increased the surface expression of the NKG2D ligand MICA in all cell lines tested, reaching statistical significance in JJN3 and RPMI-8226 cell lines. Similar results were obtained for MICB, another NKG2D ligand (Figure 2a).

We then investigated the expression of MICA and MICB after treatment with tinostamustine by RT-qPCR. In line with FCM data, tinostamustine increased the expression of *MICA* at the mRNA level in the four MM cell lines tested and that of *MICB* in all of them except for MOLP-8 (Figure 2b). We further investigated ex vivo the effect of tinostamustine on MICA and MICB expressed by MM patients’ plasma cells. In six out of the nine patients analyzed (p2188, p2249, p2316, p2400, p2483 and p2485), the expression of MICA in tinostamustine-treated cells was at least twice that in DMSO-treated cells (Figure 2c). In the case of MICB, its expression increased by more than 1.5-fold after treatment with tinostamustine in three out of the six patients analyzed (p2400, p2483 and p2485).

With respect to ULBP2 and ULBP3 (also NKG2D ligands) and CD155 and CD112 (DNAM-1 ligands), the results were heterogeneous among different MM cell lines, as shown in Table S1. Thus, among the cell lines intrinsically expressing NKG2D and DNAM-1 ligands, a trend towards an increase was observed (except for ULBP2 in MM.1S), although statistical significance was not reached in any of them.

### 2.3. Tinostamustine Enhances Daratumumab-Mediated Anti-Myeloma Activity In Vitro and Ex Vivo

**Figure 3:**
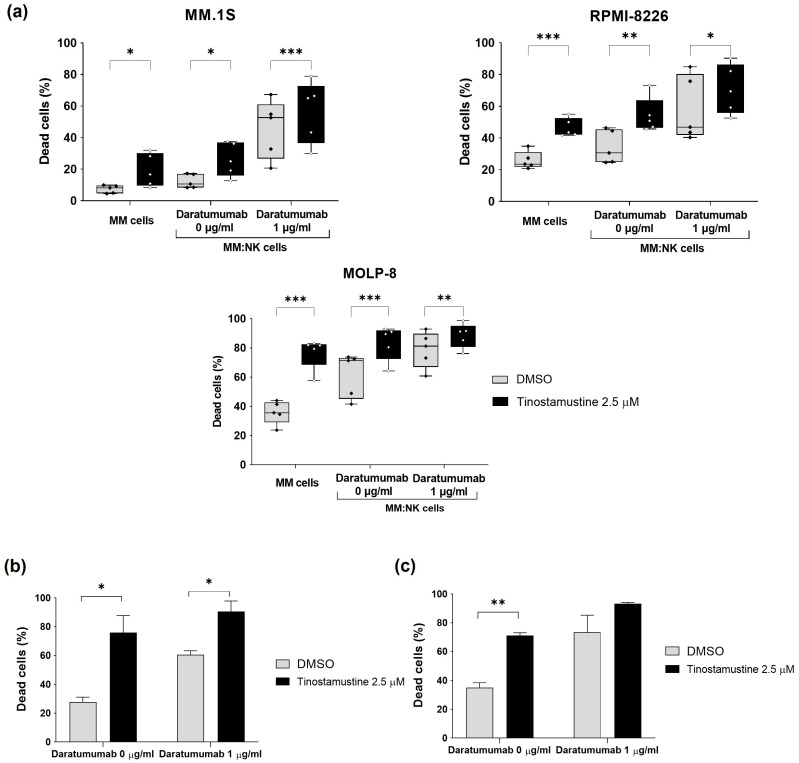
Assessment of the effect of tinostamustine in daratumumab-mediated myeloma cell death in vitro. MM cell lines were incubated with DMSO or tinostamustine (48 h). Subsequently, tinostamustine or DMSO was removed, and the corresponding daratumumab mechanism of action was assessed. (**a**) Study of daratumumab-mediated ADCC. Tinostamustine- or DMSO-pretreated myeloma cells were co-cultured with NK cells (ratio: 1:1) and incubated with daratumumab (1 μg/mL) or the isotype control (4 h). The percentage of dead cells was analyzed by FCM. Each box plot shows data from five experiments. Statistically significant differences were evaluated by the Student’s *t*-test (* *p* < 0.05, ** *p* < 0.01 and *** *p* < 0.001). (**b**) Study of daratumumab-mediated apoptosis via crosslinking. Tinostamustine- or DMSO-pretreated MOLP-8 cells were incubated with daratumumab (1 μg/mL) or the isotype control in the presence of 10 μg/mL F(ab)_2_ fragments (24 h). Apoptosis was analyzed by FCM. (**c**) Study of daratumumab-mediated CDC. Tinostamustine- or DMSO pretreated MOLP-8 cells were incubated with daratumumab (1 μg/mL) or the isotype control in the presence of 10% human serum (4 h). Cytotoxicity was analyzed by FCM. In (**b**,**c**), each bar shows the mean ± SEM (*n* = 5). Statistically significant differences were evaluated by the Student’s *t*-test (* *p* < 0.05 and ** *p* < 0.01).

Since tinostamustine upregulated CD38 and MICA/B expression in different myeloma cell lines, we wondered if this effect was translated into an improvement of daratumumab-mediated ADCC. First, pretreatment of the MM cell lines MM.1S, RPMI-8226 and MOLP-8 with tinostamustine (2.5 μM) for 48 h significantly increased the percentage of dead myeloma cells in the presence of NK cells and the absence of daratumumab (NK cell direct cytotoxicity) when compared to DMSO-pretreated cells (Figure 3a). Similarly, the percentage of dead myeloma cells was higher in tinostamustine-pretreated myeloma cells vs. DMSO-pretreated myeloma cells when they were exposed to NK cells in the presence of daratumumab (antibody-dependent cellular cytotoxicity (ADCC); Figure 3a).

Next, we evaluated whether tinostamustine could also potentiate other mechanisms of action of daratumumab, such as apoptosis via crosslinking or CDC. To confirm this hypothesis, we used MOLP-8, a MM cell line sensitive to daratumumab-mediated CDC [17] and also to daratumumab-mediated apoptosis via crosslinking (internal data from our group). Thus, tinostamustine pretreatment improved apoptosis via crosslinking in this cell line, as shown in Figure 3b; the same leaning was observed in daratumumab-mediated CDC, although statistical significance was not reached (Figure 3c).

**Figure 4:**
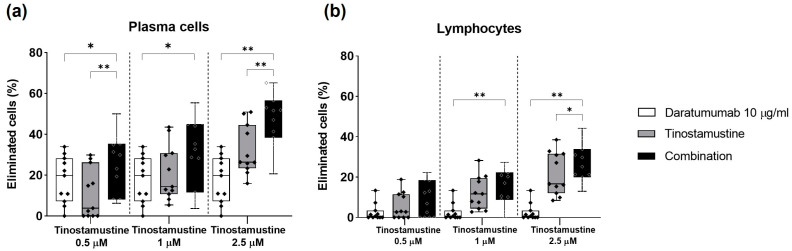
Effect of the combination of tinostamustine + daratumumab in cultures of bone marrow samples from myeloma patients. The cells were incubated for 24 h with individual treatments or the combination. The percentage of eliminated events was calculated as explained in the Materials and Methods section for myeloma cells (**a**) and lymphocytes (**b**). Each box plot shows data from 11 patients’ samples. Statistically significant differences were evaluated by Friedman’s test and Wilcoxon post hoc tests (* *p* < 0.05 and ** *p* < 0.01).

The effect of the combination of daratumumab + tinostamustine was further investigated ex vivo in freshly isolated bone marrow samples from 11 patients with MM. Our results showed that the percentage of eliminated myeloma cells was significantly higher with the combination than with individual treatments (Figure 4a), with an acceptable toxicity for the combination on healthy lymphocytes which was slightly increased with the highest doses of tinostamustine (Figure 4b).

### 2.4. Tinostamustine Enhances Daratumumab-Mediated Anti-Myeloma Activity In Vivo

**Figure 5:**
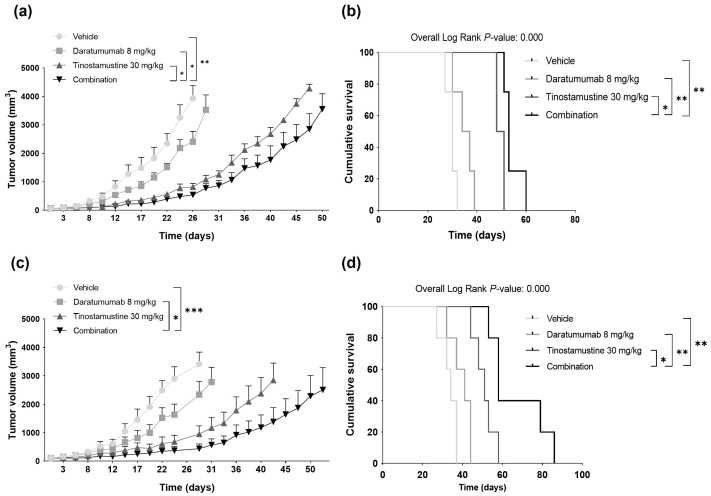
In vivo effect of the combination of tinostamustine + daratumumab in human plasmacytoma models. (**a**) NSG mice (*n* = 4/group) humanized with human NK cells from healthy donors were treated with the vehicle, daratumumab, tinostamustine or the double combination for 4 weeks, and tumor growth was monitored. (**b**) Survival of mice in (**a**) represented with a Kaplan–Meier curve. (**c**) CB17-SCID mice (*n* = 5/group) were treated with the vehicle, daratumumab, tinostamustine or the double combination for 4 weeks, and tumor growth was monitored. (**d**) Survival of mice in (**c**) represented with a Kaplan–Meier curve. Statistically significant differences in (**a**,**c**) were evaluated by one-way ANOVA and Tukey’s HSD post hoc tests (* *p* < 0.05, ** *p* < 0.01 and *** *p* < 0.001). In (**b**,**d**), statistical significance was evaluated by the log-rank (Mantel–Cox) test (* *p* < 0.05 and ** *p* < 0.01).

Finally, the in vivo activity of the combination of daratumumab + tinostamustine was evaluated in two subcutaneous plasmacytoma models. In the first study, NK cell-humanized NSG mice bearing a subcutaneous plasmacytoma derived from MM.1S cells were randomized to receive the vehicle, daratumumab, tinostamustine or the combination. As observed, the combination delayed tumor growth in comparison to individual treatments, daratumumab (*p* < 0.05) and tinostamustine (*p* < 0.05) (Figure 5a). This effect was translated into a longer median survival of mice treated with the combination (53 days ± 0.87) than mice treated with daratumumab, tinostamustine or the vehicle (34 days ± 3.5; 48 days ± NE; 30 days ± 2.5, respectively) (Figure 5b). Statistical differences in survival were found between the combination vs. the vehicle (*p* < 0.01), the combination vs. tinostamustine (*p* < 0.05) and the combination vs. daratumumab (*p* < 0.01). All treatments were generally well tolerated, with less than 10% body weight loss and a low level of severity (Figure S3 and Table S2).

In the second xenograft plasmacytoma model, CB17-SCID mice bearing a subcutaneous plasmacytoma derived from MM.1S cells were randomized to receive the vehicle, daratumumab, tinostamustine or the combination of daratumumab + tinostamustine. Treatment with the combination controlled tumor growth significantly better than daratumumab as monotherapy (*p* < 0.05) (Figure 5c). Tumor growth with the combination was slower in comparison with tinostamustine in monotherapy, although statistical significance was not reached in this case. However, in terms of survival, the combination significantly prolonged the median survival as compared to mice treated with tinostamustine in monotherapy (58 days ± 2.739 vs. 51 ± 3.286 days, respectively; *p* < 0.05) (Figure 5d). Furthermore, statistical differences in survival were also found between the combination vs. the vehicle (58 ± 2.739 vs. 34 ± 2.191 days, respectively; *p* < 0.01) and the combination vs. daratumumab (58 ± 2.739 vs. 41 ± 4.382 days, respectively; *p* < 0.01). Similarly to the NK cell-humanized NSG model, all treatment schemes were generally well tolerated, with less than 5% body weight loss and the maximum level of severity not being reached (Figure S4 and Table S3).

## 3. Discussion

Daratumumab immunotherapy has been demonstrated to be quite effective in myeloma patients, both as a single agent, with a 31.1% overall response rate (ORR) in heavily pretreated RRMM patients [36], and when combined with standards of care [4,5,6,7,8,9]. However, despite the well-established clinical efficacy of this mAb, some patients eventually become refractory to it [37]. Therefore, the modulation of molecules involved in daratumumab’s efficacy may improve the clinical outcome of the drug. In this sense, studies by other authors have demonstrated that response to daratumumab is significantly associated with levels of CD38 on tumor cells, [11,17] and that MM cells from patients at the time of progression have low CD38 expression levels [17]. According to these observations, several agents, such as all-trans retinoic acid (ATRA), immunomodulatory drugs (IMiDs), HDACis and DNMTis, may increase the expression of CD38 in myeloma cells and subsequently improve anti-CD38 treatments [11,18,19,38]. Tinostamustine, a first-in-class alkylating deacetylase inhibitor molecule, has shown anti-myeloma activity in vitro, ex vivo and in vivo, as previously reported by our group [31]. In this study, we have determined the effect of tinostamustine in increasing the expression of both CD38, the target of daratumumab, and ligands for NK cell-activating receptors on myeloma cells, and its effect in enhancing daratumumab’s efficacy in different preclinical models.

We have demonstrated that tinostamustine significantly increases the surface expression of CD38 in most of the evaluated MM cell lines. Considering that a moiety of the tinostamustine molecule has HDAC inhibitor activity [30], our data are consistent with previous observations that epigenetic drugs such as DNMT or HDAC inhibitors increase CD38 expression in myeloma cells [18,19,20]. The increase in CD38 triggered by tinostamustine seems to be, at least in part, transcriptionally regulated, since in three out of the four evaluated myeloma cell lines (JJN3, MM.1S and RPMI-8226), *CD38* mRNA levels were upregulated after treatment with tinostamustine. This effect could have been due to the increase in histone H3 acetylation in the *CD38* genomic region after treatment with tinostamustine, observed at least in the MM.1S cell line, which would allow an open conformation of the DNA favoring *CD38* transcription [39]. This finding is in line with previous data that suggested that the inhibition of HDAC6 by ricolinostat prevented the deacetylation of the *CD38* promoter and hence activated *CD38* transcription [20]. In addition to the increase in CD38 levels, we have demonstrated that tinostamustine augments the expression of several NKG2D ligands, especially MICA and MICB, in myeloma cell lines, an event that may also be beneficial when combining tinostamustine with immunotherapy treatments.

Regarding results in primary myeloma cells, tinostamustine only moderately increased CD38 in some patients with low basal expression of this molecule. Furthermore, in patients with high basal expression of CD38, tinostamustine reduced its expression, although its levels remained high compared to those patients with low basal expression. Importantly, the effect of tinostamustine on NKG2D ligands from primary myeloma cells was similar to that found in cell lines, especially with respect to MICA, since in the vast majority of samples, tinostamustine increased the expression of this ligand, including some in which CD38 decreased after tinostamustine treatment (e.g., patients 2249 and 2188).

In this study, we have demonstrated that tinostamustine increases the effect of daratumumab in vitro in MM cell lines, specifically by the mechanisms of ADCC and apoptosis via crosslinking, and also in myeloma cells from patients and in murine models. This beneficial effect exerted by tinostamustine could be due not only to the upregulation of CD38, but also to the increase in ligands for NK cell-activating receptors. In fact, as mentioned above, our results indicate that ex vivo exposure of primary myeloma cells to tinostamustine does not generally augment the levels of CD38 but is able to increase the expression of MICA and MICB. And, more importantly, the combination of tinostamustine + daratumumab has a significantly greater anti-myeloma effect than individual treatments in primary cultures. Previous works have also demonstrated that upregulation of NKG2D ligands exerted by different drugs, such as the HDACi ACY-1215 (ricolinostat) and the chemotherapeutic agent docetaxel, led to enhanced ADCC activity of mAbs [23,40]. Moreover, it has been previously demonstrated that HDACis, such as panobinostat and valproic acid (VPA), contributed to increase direct NK cell-mediated immunity against different types of tumors through induction of NKG2D ligands [21,24,41,42]. This effect has also been observed in this work, in which pretreatment with tinostamustine not only improved daratumumab-mediated ADCC, but also enhanced NK cell direct cytotoxicity over myeloma cells. Therefore, tinostamustine may be of benefit not only when combined with mAb therapy, whose efficacy depends in part on the action of NK cells, but also with other immunotherapies, such as adoptive therapy with NK cells [43,44].

In summary, our data show that tinostamustine increases the expression of CD38 as well as ligands for NKG2D in myeloma cells. These effects translate into an improvement of daratumumab’s anti-myeloma efficacy in myeloma cell lines previously exposed to tinostamustine. Furthermore, in vivo experiments confirmed that treatment with tinostamustine followed by daratumumab delayed tumor growth and improved mice survival as compared to daratumumab or tinostamustine in monotherapy. Finally, the combination of tinostamustine + daratumumab also demonstrated its efficacy in eliminating myeloma cells in ex vivo experiments performed with patients’ samples. Altogether, these data suggest that tinostamustine could be a potential candidate for improving the anti-myeloma efficacy of anti-CD38 mAbs, such as daratumumab or isatuximab, in the clinical setting. Nevertheless, future preclinical and clinical studies will determine the best time schedule for the administration of tinostamustine and daratumumab or other anti-CD38 mAbs, as well as a more precise positioning of these combinations within the existing therapy landscape of MM.

## 4. Materials and Methods

### 4.1. Drugs and Reagents

Tinostamustine was provided by Mundipharma Research Limited (Basel, Switzerland), and daratumumab was obtained from the Pharmacy Department of the University Hospital of Salamanca (Salamanca, Spain). Daratumumab isotype control IgG1 was purchased from Sigma-Aldrich (San Luis, MO, USA). Cell culture media, fetal bovine serum and penicillin–streptomycin were purchased from the Invitrogen Corporation (Gaithersburg, MD, USA).

### 4.2. MM Cell Lines, Patient Samples and Cultures

The origin, authentication and in vitro culture methods of MM cell lines have been previously reported [45]. BM samples from MM patients were obtained following approval from the University Hospital of Salamanca Ethical Committee (CEIm ref.: 2021/06/799) and written informed consent from patients. Research with human samples was conducted in accordance with the Declaration of Helsinki guidelines. Table S4 shows the disease status of each patient and the type of study in which each sample was used.

### 4.3. Flow Cytometry (FCM)

Mouse anti-human CD138-FITC, CD56-PE, CD45-PerCP-Cy5.5 and CD38-APC and the IgG1 κ-APC isotype control were obtained from BD Biosciences (San Jose, CA, USA). Mouse anti-human MICA-Alexa Fluor 488, MICB-Alexa Fluor 488, ULBP3-PE, CD155-APC (PVR), CD112-APC (Nectin-2) and the corresponding isotype controls IgG2B-Alexa Fluor 488 and IgG2A-PE were purchased from R&D Systems (Minneapolis, MN, USA). Mouse anti-human ULBP-2 and anti-mouse-Alexa Fluor 488 were obtained from Sigma-Aldrich. CD38me-FITC (anti-human CD38 multi-epitope) was acquired from Cytognos (Salamanca, Spain), and CD45-APC was acquired from Immunostep (Salamanca, Spain).

For surface staining, cells were incubated with the appropriate antibodies and 7AAD (Immunostep) for 15 min in the dark. Data were acquired using a FACSCalibur cytometer (BD Biosciences) and analyzed using Infinicyt^TM^ software 1.7 version (Cytognos). Specifically, the expression of CD38 or ligands for NK cell-activating receptors (MICA, MICB, ULBP2-3, PVR and CD112) in MM cell lines was determined in the viable cell population (7AAD^−^). In primary myeloma cells, the expression of CD38 was analyzed over the viable myeloma population using the panel CD138-FITC/CD56-PE/7AAD/CD38-APC, whereas the MICA/B-AF488/CD56-PE/7AAD/CD38-APC panel was used to determine MICA/B expression. Normalized mean fluorescence intensity (MFI) was calculated by dividing the median fluorescence obtained with the specific antibody by the median fluorescence of the corresponding isotype control.

### 4.4. Real-Time Quantitative PCR (RT-qPCR)

Total RNA was isolated using the RNeasy Mini Kit (Qiagen, Hilden, Germany). Following reverse transcription, PCR was performed using TaqMan Gene Expression Assays (Applied Biosystems, Foster City, CA, USA): CD38 (Hs01120071_m1), MICA (Hs00741286_m1), MICB (Hs00792952_m1) and GAPDH (Hs99999905_m1). The results were expressed as the fold change relative to the expression of the target gene in the control cells, using endogenous expression of GAPDH for normalization.

### 4.5. Chromatin Immunoprecipitation (ChIP)

RPMI-8226 and MM.1S cell lines were treated with DMSO or tinostamustine (2.5 μM) for 48 h. Then, the cells were fixed with 1% of Pierce™ fresh methanol-free formaldehyde (ThermoFisher, Waltham, MA, USA) in PBS for 15 min at room temperature and prepared for sonication with the truChIP Chromatin Shearing Kit (Covaris, Woburn, MA, USA), following the manufacturer’s instructions and as previously described [46].

For ChIP-qPCR, the immunoprecipitated DNA for each sample was diluted 1/10. Then, 4 μL of diluted DNA and specific primers (Table S5) were used for each reaction. RT-qPCR was performed in technical triplicates for each biological replicate, using LightCycler^®^ 480 SYBR Green Mix (Roche, Basel, Switzerland). The relative amount of immunoprecipitated DNA was compared to the input DNA for each condition.

### 4.6. Evaluation of Daratumumab Binding to Myeloma Cells

To evaluate daratumumab binding to myeloma cells, MM cell lines were first pretreated with tinostamustine (2.5 μM) or DMSO for 48 h. Then, cells were washed to remove tinostamustine or DMSO from the culture medium, and daratumumab (10 μg/mL) was added and incubated for 30 min. After that time, cells were washed with PBS twice and incubated for 30 additional minutes with anti-human-IgG1-AlexaFluor488 (Invitrogen, Carlsbad, CA, USA). Cells were acquired in a FACSCalibur cytometer, and normalized MFI was calculated as described above.

### 4.7. ADCC, CDC and Apoptosis via Crosslinking

To assess the effect of tinostamustine pretreatment on cytotoxic mechanisms mediated by daratumumab, MM cell lines were first incubated in the presence of tinostamustine (2.5 μM) or DMSO for 48 h. Cells were then washed to remove tinostamustine or DMSO from the culture medium, and the corresponding cytotoxic assay (ADCC, CDC or apoptosis via crosslinking) was performed.

For the evaluation of ADCC, tinostamustine- or DMSO-pretreated cells were co-cultured with NK cells obtained from healthy donors in the presence of daratumumab (1 μg/mL) or the corresponding isotype control for 4 h (ratio: 1:1). Briefly, peripheral blood mononuclear cells (PBMCs) were isolated from healthy donor buffy coats by Lymphoprep^TM^ (STEMCELL Technologies, Saint Égrève, France) density gradient centrifugation. Afterwards, NK cells were isolated from PBMCs using the NK Cell Isolation Kit (Miltenyi Biotec, Bergisch Gladbach, Germany) in an autoMACS^®^ Pro Separator (Miltenyi Biotec), following the manufacturer’s instructions.

For CDC analysis, pretreated MM cells were cultured for 4 h in the presence of daratumumab (1 μg/mL) or the corresponding isotype control plus 10% human serum as a complement source.

To evaluate apoptosis via crosslinking, pretreated MM cells were incubated for 24 h with daratumumab (1 μg/mL) or the corresponding isotype control plus 10 μg/mL F(ab)_2_ fragments (Jackson ImmunoResearch Laboratories, Inc., Ely, Cambridgeshire, UK) for the induction of crosslinking.

In all three assays, the cytotoxic effect on MM cells was evaluated by FCM with Annexin V/7AAD staining (Immunostep) after treatment with daratumumab. Specifically, for ADCC, additional staining with CD38me and CD45 was included to identify MM cells (CD38^+^ CD45^−^) in the NK cell/myeloma co-culture.

### 4.8. Ex Vivo Analysis of Apoptosis in BM Samples from Myeloma Patients

BM samples from myeloma patients were lysed to remove red blood cells and cultured, as previously described [47], in the presence of daratumumab (10 μg/mL), tinostamustine (0.5, 1 and 2.5 μM), the combination of daratumumab + tinostamustine or daratumumab’s isotype control for 24 h. After the incubation period, cells were collected in BD Trucount™ Absolute Counting Tubes (Becton Dickinson, Franklin Lakes, NJ, USA) and stained with the corresponding antibodies (CD38me-FITC/CD45-PerCP-Cy5.5/CD56-PE) for FCM analysis. The percentage of eliminated cells was calculated following the manufacturer´s instructions for myelomatous plasma cells (CD38^+bright^, CD45^−/low^, SSC^low/intermediate^ and CD56^−/+^) and normal lymphocytes (CD45^++^ and SSC^low^), as described elsewhere [48].

### 4.9. In Vivo Evaluation of the Efficacy of the Combination of Tinostamustine + Daratumumab in Subcutaneous Plasmacytoma Models

For the first subcutaneous human plasmacytoma model, NOD.Cg-Prkdc^scid^Il2rg^tm1Wji/SzJ^ (NSG) mice (16 female mice, 5–6 weeks old; obtained from Servicio de Experimentación Animal O.M.G., USAL, Salamanca, Spain) were subcutaneously injected with 3 × 10^6^ MM.1S cells in 100 μL RPMI-1640 medium + 100 μL Matrigel (BD Biosciences) into the right flank. When tumors became palpable, the mice were randomized into 4 groups (4 mice/group) to receive one of the following treatments: tinostamustine (30 mg/kg, i.v., weekly), daratumumab (8 mg/kg, i.p., weekly), the combination or the vehicle [PBS vehicle solution with 0.4 mg/kg daratumumab isotype control IgG1, 15% 2-hydroxypropyl-β-cyclodextrin (HPBCD), 1.5% acetic acid and 1.25% NaHCO_3_]. Tinostamustine was administered 24 h before daratumumab as appropriate. In addition, all mice were i.v. injected with human NK cells isolated from healthy donor buffy coats as described above (1.4–2 × 10^6^/mouse/weekly, depending on NK cell availability in each buffy coat), coinciding with the daratumumab dosing regimen. Both human NK cells and the treatments were administered during 4 consecutive weeks. Caliper measurements of tumor diameters were performed three times a week, and the tumor volume was estimated as the volume of a 3D ellipse using the following formula: V = 4/3 π × (a/2) × (b/2)^2^, where “a” and “b” correspond to the longest and shortest diameter, respectively. Animals were sacrificed when tumors reached 2 cm as the longest diameter or if distress signs were observed. Statistical differences in tumor volumes between the different groups were evaluated using one-way analysis of variance (ANOVA) and Tukey’s HSD post hoc tests.

For the second subcutaneous human plasmacytoma model, CB17-SCID mice (20 female mice, 5–6 weeks old; obtained from The Jackson Laboratory, Bar Harbor, ME, USA) were used. The design of the experiment (treatment doses and time schedule) was identical to the one performed with NSG mice except for the inoculation of human NK cells, since CB17-SCID mice hold their own functional NK cells [49].

Animal experiments were conducted according to institutional guidelines for the use of laboratory animals and after permission was granted by the University of Salamanca Animal Ethical Committee for animal experimentation and the Agriculture and Livestock Council of Junta de Castilla y León (registry numbers: 0000061 and 292; Registered User Center: ES372740000046).

### 4.10. Statistical Analyses

Statistical analyses were carried out using GraphPad Prism software v6 (GraphPad Software, San Diego, CA, USA), as indicated for each experiment. Otherwise specified, data are summarized as the mean ± SEM. The Student’s *t*-test or ANOVA tests were used to determine statistical significance. *p*-values lower than 0.05 were considered statistically significant.

## Data Availability

The datasets used and/or analyzed during the current study are available from the corresponding author on reasonable request.

## Supplementary Materials

The following supporting information can be downloaded at: https://www.mdpi.com/article/10.3390/ijms25094718/s1.
