# Supplementary material for: Tinostamustine (EDO-S101), an Alkylating Deacetylase Inhibitor, Enhances the Efficacy of Daratumumab in Multiple Myeloma by Upregulation of CD38 and NKG2D Ligands
Source: Int J Mol Sci. 2024 Apr 26;25(9):4718. doi: 10.3390/ijms25094718 (PMC11083018; doi:10.3390/ijms25094718)
Supplement: Supplementary file 1 [file ijms-25-04718-s001.zip › ijms-2940463-supplementary.pdf]

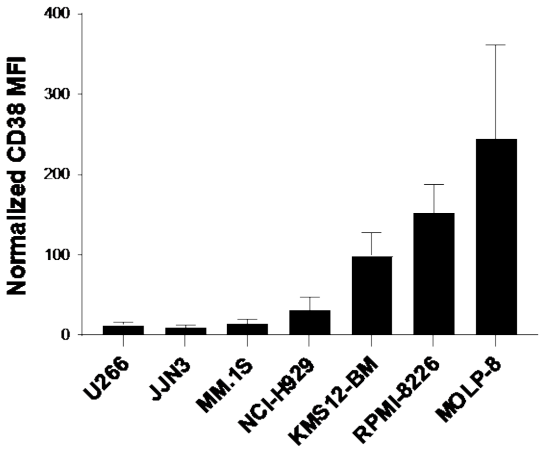

Figure S1. Normalized MFI expression of CD38 on myeloma cell lines analyzed by flow cytometry.

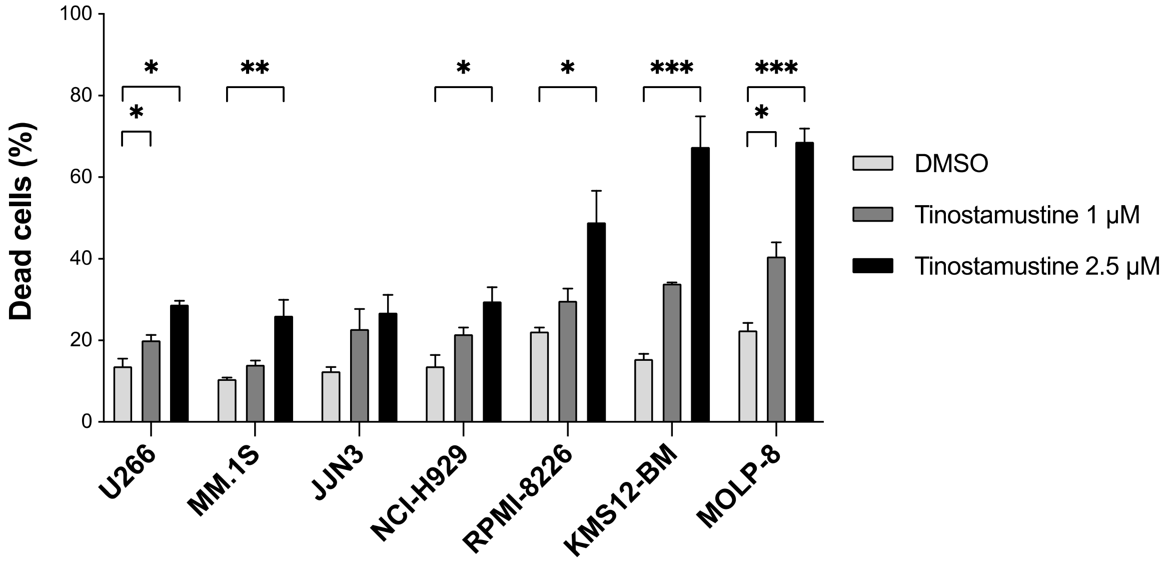

Figure S2. Evaluation of the cytotoxic effect of tinostamustine in MM cell lines. The indicated MM cell lines were incubated with increasing doses of tinostamustine for 48 hours. Cytotoxicity was analyzed by flow cytometry after staining with Annexin V/7AAD. Each bar shows mean ± SEM (n=3). Statistically significant differences were evaluated by one-way ANOVA followed by Tukey's HSD post-hoc test (*p<0.05; **p<0.01; ***p<0.001).

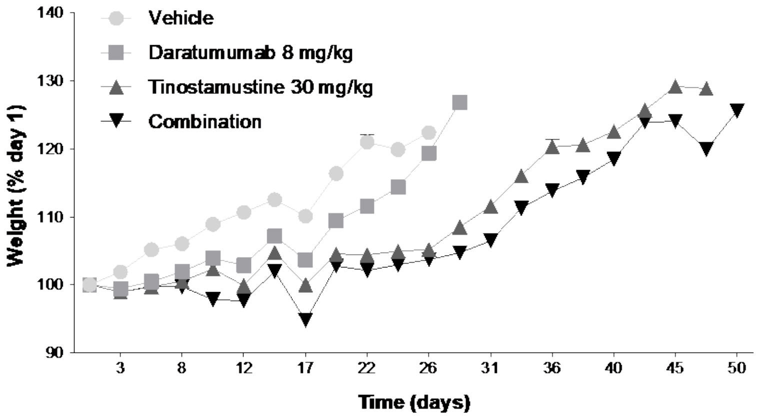

Figure S3. Effect of the administration of each treatment on body weight of NK cell-humanized NSG mice. The body weight of mice was monitored throughout the treatment period, and the percentage of weight at each time point was calculated considering day 1 as 100%. X-axes express the time in days from the beginning of the treatment.

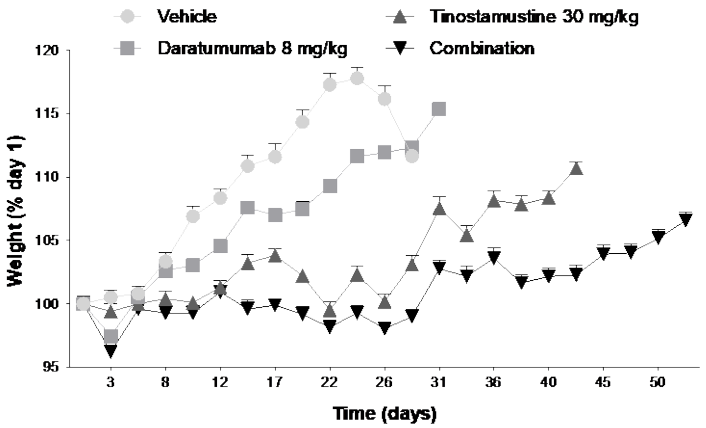

Figure S4. Effect of the administration of each treatment on body weight of CB17-SCID mice. The body weight of mice was monitored throughout the treatment period, and the percentage of weight at each time point was calculated considering day 1 as 100%. X-axes express the time in days from the beginning of the treatment.

Table S1. Effect of tinostamustine on the expression of several ligands for NKG2D and DNAM-1 as assessed by flow cytometry. Median fluorescence intensity (MFI) levels of the indicated ligands normalized to isotype control after treatment with tinostamustine (1 and 2.5 μM) for 48 hours. Results are shown as the percentage with respect to the expression in DMSO-treated cells, which was considered as 100%. Expression levels correspond to the average of three experiments. Absence of expression is indicated as "-".

	Tinostamustine (μM)	ULBP2	ULBP3	CD155	CD112
JJN3	0	100	-	100	-
	1	110.5±9.5	-	161.4±14.3	-
	2.5	112.9±13.4	-	156.8±24.7	-
MM.1S	0	100	-	100	-
	1	95.8±4.5	-	113.3±3.9	-
	2.5	96.3±12.3	-	111.5±15.3	-
RPMI-8226	0	-	100	100	100
	1	-	111.7±14.8	123.9±6.1	116.6±13.2
	2.5	-	129.6±20.2	168.2±24.8	142.8±11.5
MOLP-8	0	100	-	-	-
	1	116.1±4.6	-	-	-
	2.5	124.7±13.5	-	-	-

Table S2. Severity observed in the in vivo study with the human plasmacytoma model in NK cell-humanized NSG mice. The toxicity/distress of the mice was evaluated according to different parameters: state of the fur (in blue), state of the tail (no alterations observed), paralysis (no alterations observed) and ulceration (in purple). Moreover, each parameter was evaluated in a range of severity from 1 to 4, being 1 the less severe and 4 the most severe. In case of a mouse showing a severity of 4 (either with one parameter or summing up different parameters) the mouse was sacrificed. The cells in grey show the day each mouse died.

		Time (days)																							
Treatment group	Mouse	1	2	8	9	15	16	23	24	26	29	31	33	36	38	40	43	45	47	50	52	54	57	59	60
Control	C02M00																								
	C02M01																								
	C02M03																								
	C02M10																								
Daratumumab 8 mg/kg	C03M00																								
	C03M01																								
	C03M03																								
	C03M10																								
Tinostamustine 30 mg/kg	C04M00																								
	C04M01																								
	C04M03					1	1	1	1	1	1	1	1	1	1	1	1	1	1						
	C04M10																								
Combination	C05M00																								
	C05M01										1	1	1	1	1	1	1	1	1	1					
	C05M03											1	1	1	1	1	1	1	1	1	1				
	C05M10																								

Table S3. Severity observed in the *in vivo* study with the human plasmacytoma model in CB17-SCID mice. The toxicity/distress of the mice was evaluated according to different parameters: state of the fur (no alterations observed), state of the tail (no alterations observed), paralysis (no alterations observed) and ulceration (in purple). Moreover, each parameter was evaluated in a range of severity from 1 to 4, being 1 the less severe and 4 the most severe. In case of a mouse showing a severity of 4 (either with one parameter or summing up different parameters), the mouse was sacrificed. The cells in grey show the day each mouse died.

		Time (days)																																				
Treatment group	Mouse	1	2	8	9	16	17	20	23	24	25	27	30	32	34	37	39	41	44	46	48	51	53	55	58	60	62	65	67	69	72	74	76	79	81	83	86	87
Vehicle	C01M00												■																									
	C01M01								1	1	1	2	2	2	■																							
	C01M03								1	1	2	2	2	2	3	■																						
	C01M10														1	3	■																					
	C01M30												2	2	2	2	■																					
Daratumumab 8 mg/kg	C02M00											1	1	1	■																							
	C02M01							1	1	1	1	1	1	1	1	1	1	1	1	■																		
	C02M03										1	1	1	1	1	1	■																					
	C02M10										1	2	2	2	2	2	2	2	■																			
	C02M30																			■																		
Tinostamustine 30 mg/kg	C03M00													1	1	1	1	1	1	■																		
	C03M01													1	2	2	2	2	2	2	2	■																
	C03M03																					2	■															
	C03M10																							■														
	C03M30																			2	2	2	2	2	2	■												
Combination	C04M00																									■												
	C04M01																																		■			
	C04M03																		1	1	1	1	1	■														
	C04M10																										■											
	C04M30																																					■

Table S4. Patient samples used in the studies. The table shows the disease status of each patient. The type of study in which each sample was used is indicated in grey-filled cells.

Patient code	Disease status	Analysis of CD38 (Fig 1d)	Analysis of MICA (Fig 2c)	Analysis of MICB (Fig 2c)	Analysis of drugs (Fig 4)
p2043	Progressive MM				
p2048	Progressive MM				
p2049	Newly diagnosed MM				
p2053	Relapsed MM				
p2071	Newly diagnosed MM				
p2072	Newly diagnosed MM				
p2121	Newly diagnosed MM				
p2134	Newly diagnosed MM				
p2135	Progressive MM				
p2188	Newly diagnosed MM				
p2189	Relapsed MM				
p2229	Relapsed MM				
p2246	Relapsed MM				
p2249	Progressive MM				
p2316	Newly diagnosed MM				
p2400	Progressive MM				
p2480	Newly diagnosed MM				
p2483	Newly diagnosed MM				
p2485	Relapsed MM				

Table S5. Primers used for ChIP-PCR. GAPDH gene was used as a positive control for the histone 3 acetylation (H3Ac), whereas SAT2 gene was used as a negative control for the same histone modification. F letter stands for forward primer and R letter for reverse.

Gene	Primers used (5' → 3')
CD38	F: CCTCGCTTTCACCGGGAAAT
	R: TGCGGGATTTTGCTATCCCA
GAPDH	F: TACTAGCGGTTTTACGGGCG
	R: TCGAACAGGAGGAGCAGAGAGCGA
SAT2	F: CATCGAATGGAAATGAAAGGAGTC
	R: ACCATTGGATGATTGCGATCAA
